# Combining niche shift and population genetic analyses predicts rapid phenotypic evolution during invasion

**DOI:** 10.1111/eva.12592

**Published:** 2018-02-02

**Authors:** Erik E. Sotka, Aaron W. Baumgardner, Paige M. Bippus, Christophe Destombe, Elizabeth A. Duermit, Hikaru Endo, Ben A. Flanagan, Mits Kamiya, Lauren E. Lees, Courtney J. Murren, Masahiro Nakaoka, Sarah J. Shainker, Allan E. Strand, Ryuta Terada, Myriam Valero, Florian Weinberger, Stacy A. Krueger‐Hadfield

**Affiliations:** ^1^ Department of Biology College of Charleston Charleston SC USA; ^2^ UMI EBEA 3614, CNRS Sorbonne Universités UPMC, UCCh, UACH Station Biologique de Roscoff Roscoff France; ^3^ United Graduate School of Agricultural Sciences Kagoshima University Kagoshima Japan; ^4^ Faculty of Marine Bioscience Fukui Prefectural University Obama Fukui Japan; ^5^ Akkeshi Marine Station Field Science Center for Northern Biosphere Hokkaido University Hokkaido Japan; ^6^ Helmholtz‐Zentrum für Ozeanforschung Kiel (GEOMAR) Kiel Germany; ^7^ Department of Biology University of Alabama at Birmingham Birmingham AL USA

**Keywords:** biological invasions, genetic adaptation, heat tolerance, latitudinal cline, niche shift, Rhodophyta

## Abstract

The rapid evolution of non‐native species can facilitate invasion success, but recent reviews indicate that such microevolution rarely yields expansion of the climatic niche in the introduced habitats. However, because some invasions originate from a geographically restricted portion of the native species range and its climatic niche, it is possible that the frequency, direction, and magnitude of phenotypic evolution during invasion have been underestimated. We explored the utility of niche shift analyses in the red seaweed *Gracilaria vermiculophylla*, which expanded its range from the northeastern coastline of Japan to North America, Europe, and northwestern Africa within the last 100 years. A genetically informed climatic niche shift analysis indicates that native source populations occur in colder and highly seasonal habitats, while most non‐native populations typically occur in warmer, less seasonal habitats. This climatic niche expansion predicts that non‐native populations evolved greater tolerance for elevated heat conditions relative to native source populations. We assayed 935 field‐collected and 325 common‐garden thalli from 40 locations, and as predicted, non‐native populations had greater tolerance for ecologically relevant extreme heat (40°C) than did Japanese source populations. Non‐native populations also had greater tolerance for cold and low‐salinity stresses relative to source populations. The importance of local adaptation to warm temperatures during invasion was reinforced by evolution of parallel clines: Populations from warmer, lower‐latitude estuaries had greater heat tolerance than did populations from colder, higher‐latitude estuaries in both Japan and eastern North America. We conclude that rapid evolution plays an important role in facilitating the invasion success of this and perhaps other non‐native marine species. Genetically informed ecological niche analyses readily generate clear predictions of phenotypic shifts during invasions and may help to resolve debate over the frequency of niche conservatism versus rapid adaptation during invasion.

## 1 INTRODUCTION

Non‐native species homogenize the Earth's biota and profoundly alter local community processes and ecosystem function (Lockwood, Hoopes, & Marchetti, 2007; Maggi et al., 2015; Vila et al., 2011). The successful establishment of non‐native populations can be facilitated by microevolution (Allendorf & Lundquist, 2003; Colautti & Lau, 2015; Cox, 2004) through several mechanisms. Before invasion occurs, local adaptation across the native geographic range may yield a subset of populations that perform well in non‐native habitats (Hufbauer et al., 2012; e.g., Bossdorf, Lipowsky, & Prati, 2008 and Rey, Estoup, Vonshak, Loiseau, & Chifflet, 2012). Evolutionary shifts that occur at the site of initial introduction (termed bridgehead effect; Lombaert et al., 2010) yield lineages which subsequently spread throughout non‐native territories. Postintroduction adaptation arises when evolutionary changes occur in multiple locations after arrival in a novel habitat (Lee, 2015; Moran & Alexander, 2014), sometimes through genetic mixture of multiple distinct sources (e.g., Lavergne & Molofsky, 2007). Alternatively, it is also possible that no adaptation has occurred, as when plastic or canalized phenotypes allow successful establishment in non‐native habitats (e.g., Geng et al., 2007; Parker, Rodriguez, & Loik, 2003).

**Figure 1:**
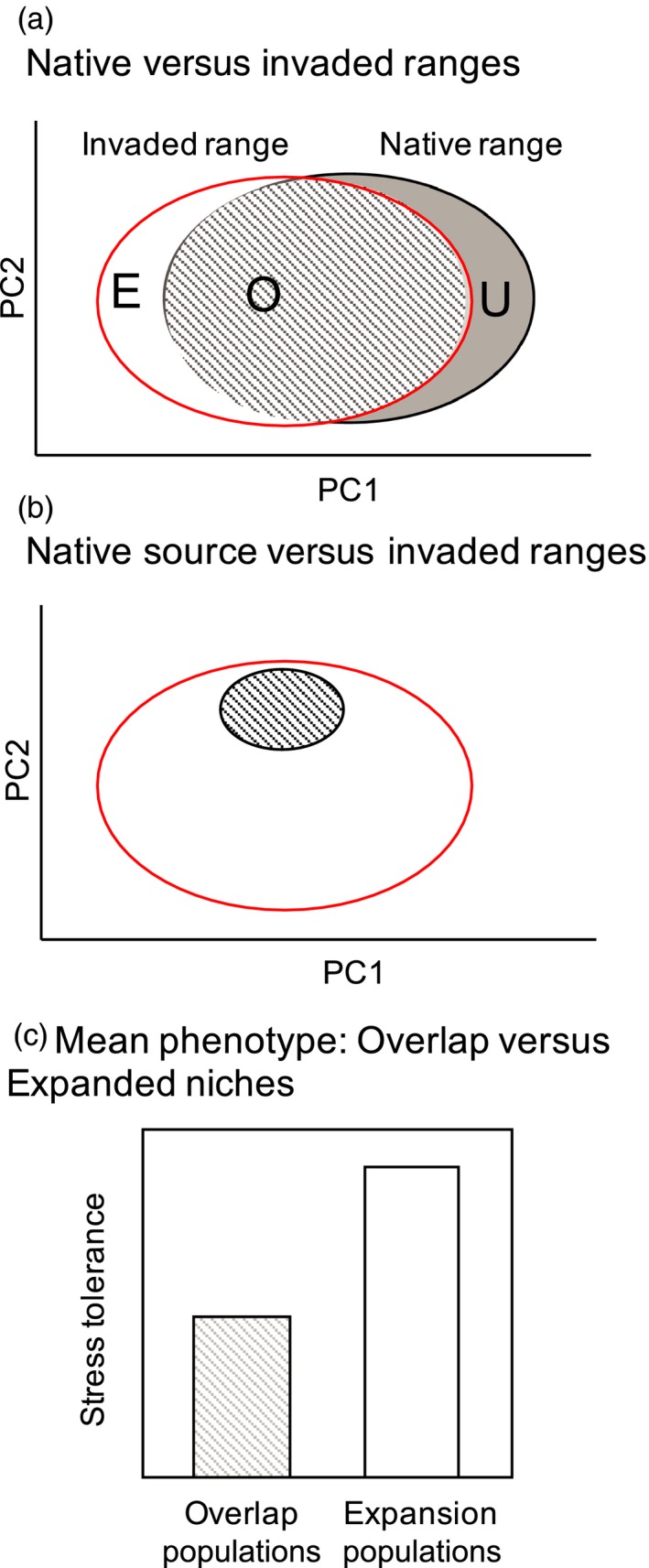
Combining niche shift analysis (Guisan et al., 2014) and population genetics predicts rapid phenotypic evolution during invasion. (a) The environmental niche space for native (gray circle) versus invaded (red outline) ranges is presented, along with Overlap niches (O; hatched), Unfilled niches (U), and Expanded niches (E; see text for definitions). (b) When the invasion is sourced from a subset of the native range, the niche shift from source (hatched) to invaded regions (white) differs from when the niche is sourced from the subset of the entire native range. (c) Phenotypic shifts (e.g., stress tolerance) are inferred by differences among populations in the Overlap versus Expansion niches

While a growing number of studies have identified shifts in ecologically relevant phenotypes, including tolerance for abiotic stresses, several recent reviews indicate that most non‐native species move into similar climatic niches in the non‐native range (Peterson, 2011; Petitpierre et al., 2012). This is potentially paradoxical, as niche conservatism implies that evolutionary change is relatively rare (Guisan, Petitpierre, Broennimann, Daehler, & Kueffer, 2014). However, these analyses may underestimate the frequency of niche shift and by extension, adaptive evolution when they do not account for invasion sources (Figure 1; Ikeda et al., 2016). Just as identification of the source populations improves the ability to detect adaptive versus neutral shifts in phenotype (Colautti & Lau, 2015; Keller & Taylor, 2008), incorporating genetics and use in identification of source populations into niche analyses should improve the detection of true niche shifts between native source and non‐native populations.

Here, we use niche analysis to generate testable predictions of microevolutionary shifts in a phenotype (i.e., abiotic stress tolerance). Our approach (Figure 1) follows that of Broennimann et al., 2012; and Guisan et al., 2014;. First, we evaluate the overlap (“Overlap” or O) of the climatic niches of populations in the native and invasive ranges. We then evaluate the phenotypes of populations of invaded habitats that are outside the climatic niches of the populations of the native range (“Expanded” or E). In cases where the genetic origin of the invasion can be traced back to a subset of the populations from the native range, we can more accurately identify niche shifts and phenotypic shifts between the native source and invaded ranges. This genetically informed niche shift (Figure 1b) may profoundly differ from the niche shifts inferred between the entirety of the native and invaded ranges (Figure 1a) which is typically how these analyses are performed (e.g., Petitpierre et al., 2012).

The haplodiplontic red seaweed *Gracilaria vermiculophylla* (Ohmi) Papenfuss is native to the northwestern Pacific Ocean (Terada & Yamamoto, 2002), but is now common to high‐salinity estuaries of northwestern Africa, Europe, and both coastlines of North America (Krueger‐Hadfield et al., 2017). *G. vermiculophylla* is an ecosystem engineer that when established in a site can cover up to 80%–100% of soft‐sediment habitats, outcompete native macroalgae, and alter community structure, species interactions, trophic pathways, and nutrient cycling (Byers, Gribben, Yeager, & Sotka, 2012; Gonzalez, Smyth, Piehler, & McGlathery, 2013; Kollars, Byers, & Sotka, 2016; Thomsen, McGlathery, Schwarzschild, & Silliman, 2009). The population ecology of *G. vermiculophylla* differs profoundly between native and non‐native regions. Native populations are nearly always attached to pebbles and rocks either on wave‐exposed or estuarine habitats while nearly all non‐native populations occur on low‐energy estuarine mudflats drifting as unattached thalli or glued onto tubes by decorator worms (Kollars et al., 2016; Krueger‐Hadfield et al., 2016). This shift from primarily attached to unattached thalli is correlated with shifts from sexual to asexual reproduction and slight to strong diploid bias within populations (Krueger‐Hadfield et al., 2016).

**Figure 2:**
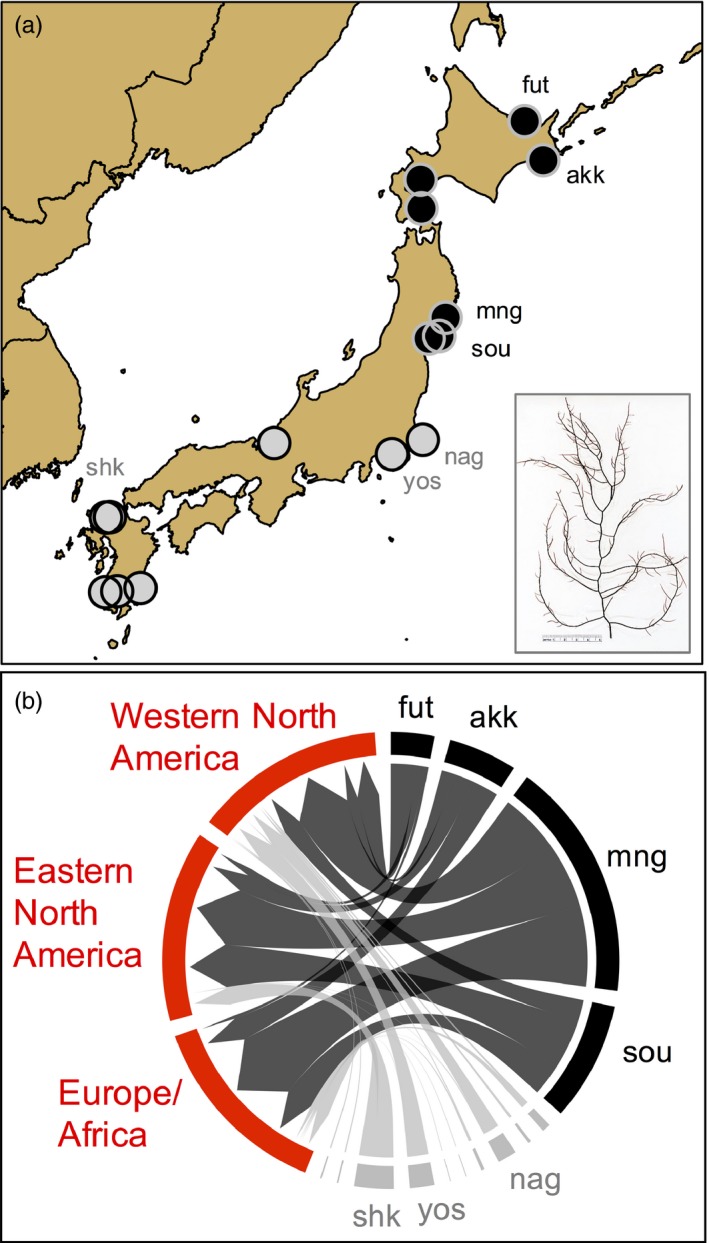
Genetic identification of source populations in northeastern Japan, as reported by Krueger‐Hadfield et al., 2017. (a) A map showing Japanese source (black dots) and nonsource populations (gray dots) sampled for this study. These are a subset of the populations genotyped and reported in Krueger‐Hadfield et al., 2017. (b) A DAPC‐based assignment of microsatellite genotypes in the introduced range to those of native populations (modified from Figure S6 of Krueger‐Hadfield et al., 2017). The figure was generated using *R::circlize* (Gu, Gu, Eils, Schlesner, & Brors, 2014)

In a previous study, we identified the Pacific coastline of northeastern Japan as the principal source region of the invasion using mitochondrial and microsatellite genotypes of over 2,500 thalli from 53 non‐native populations and 37 native populations in China, South Korea, and Japan (Krueger‐Hadfield et al., 2017). Multiple analyses indicate that four populations (fut, akk, mng, and sou) sourced the vast majority of thalli in the non‐native range. For example, a Bayesian genetic clustering algorithm of microsatellite genotypes (*InStruct* Gao, Williamson, & Bustamante, 2007) indicated that 95%–99% of non‐native populations are assigned to the genetic clusters 1, 4, and 5 (k = 5) which dominate these four populations. The exception occurs in northwestern United States and British Columbia, where ~75% of the thalli were dominated by these same genetic clusters. In Figure 2, we present results from a DAPC‐based assignment protocol (Jombart, Devillard, & Balloux, 2010; modified from Figure S6 of Krueger‐Hadfield et al., 2017), which also points to these same populations as the principal source for introduced populations.

The identification of a source region directly informs our phenotypic analyses in two ways. We compared phenotypes of northeastern Japan against those in the introduced range to specifically assess the role of rapid evolution during and/or after introduction from those that share population genetic history. We also used the genetically informed niche overlap analysis to predict variation in stress tolerance.

## 2 MATERIALS AND METHODS

### 2.1 Niche shift analyses

We define niche shifts as a change in the realized niche that reflects microevolutionary changes in the fundamental niche of a population, but recognize that niche shifts may also reflect changes in biotic interactions or dispersal limitation (Guisan et al., 2014; Marcelino & Verbruggen, 2015; Petitpierre et al., 2012). There is reason to suspect that biotic interactions do not strongly limit invasive success of *G. vermiculophylla. G. vermiculophylla* is less palatable than co‐occurring seaweeds to consumers in its non‐native range (Kollars et al., 2016; Nejrup, Pedersen, & Vinzent, 2012), presumably because of elevated prostaglandin‐based defenses (Hammann, Wang, Rickert, Boo, & Weinberger, 2013; Nylund, Weinberger, Rempt, & Pohnert, 2011). Moreover, intertidal, high‐salinity mudflats where *G. vermiculophylla* resides have relatively few competitors (Byers et al., 2012; Thomsen et al., 2009).

There are two related approaches used to identify niche shifts and infer adaptive phenotypic shifts (Guisan et al., 2014). Environmental niche models compare the overlap of the geographic distributions of habitat in native and non‐native ranges (Jiménez‐Valverde et al., 2011; Warren, Glor, & Turelli, 2008). The approach we use is an ordination model, which generates a multivariate description of the environmental characteristics where species occur. A previous study demonstrated that the ordination model quantified niche shifts and conservatism with more accuracy relative to environmental niche models (Broennimann et al., 2012).

We followed the approaches of Broennimann et al. (2012) and Guisan et al. (2014) to assess the relative niches of the potential and realized niche space of native and non‐native populations. We compiled 193 locations for which *G. vermiculophylla* was collected and identified using molecular markers (Appendix A); 153 of these occurrences are from Kim, Weinberger, & Boo, 2010; and Krueger‐Hadfield et al., 2017. We did not include the warm low‐latitude Mediterranean estuaries of Italy for further analyses (e.g., Sfriso, Wolf, Maistro, Sciuto, & Moro, 2012) because we did not genotype nor phenotype their thalli.

**Figure 3:**
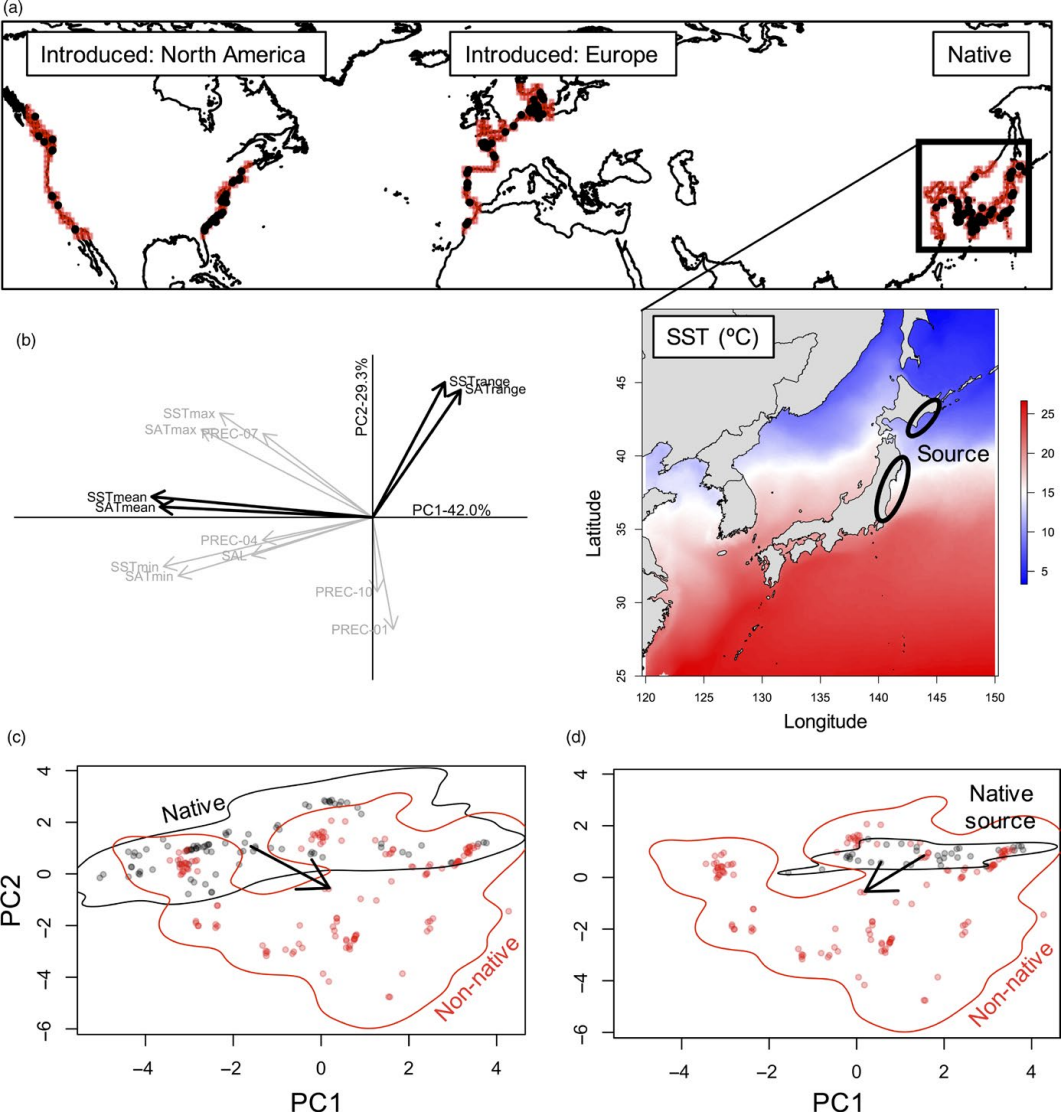
Niche shift model for *Gracilaria vermiculophylla*. (a) Potential habitat (pink dots) and occurrences (black dots) of *G. vermicullophylla*. Inset: Mean sea surface temperature (°C) of northeastern Pacific waters. Northeastern Japan is the source of the invasion (black circles). (b) Environmental variables correlated with principal component axes. Variables in bold are the two variables that best explain axes 1 and 2. (c) Niche Shift model for non‐native populations versus all native populations, and (d) for non‐native populations versus the source region. Solid 99% kernel density estimate indicates the PCA space for potential habitat, and arrows indicate the shift in centroids of realized niche space

We assumed that the species’ current distribution in the native range reflects all suitable habitats in its native range. Further, we assume the potential habitat of *G. vermiculophylla* (sensu Broennimann et al., 2012) is the coastlines that lie between the northern and southern ends of the latitudinal range of the current distribution in North America, Europe, and northwestern Africa (Figure 3a). This approach is conservative because it likely underestimates the potential habitat in the non‐native range. To quantify potential habitat points, we downloaded a 10‐m resolution shapefile of the coastline from http://www.naturalearthdata.com/, based on the World Data Bank dataset. We generated a grid of 1ºx1º squares that cover the earth and kept the center point of those grids that overlap with any coastline using *spatstat* and *rgdal* (Baddeley, Rubak, & Turner, 2015; Bivand, Keitt, & Rowlingson, 2017).

For each occurrence and potential habitat location, we identified the mean, maximum and minimum mean sea surface temperature and surface air temperature, and yearly ocean salinity values from *BioOracle* (Tyberghein et al., 2012) and the monthly precipitation means from *WorldClim* (Hijmans, Cameron, Parra, Jones, & Jarvis, 2005). Due to the difficulty in generating both ocean and terrestrial data points, we collected fifty randomly generated points within a 50 km radius of each occurrence point using *raster* and *sp* (Bivand, Pebesma, & Gomez‐Rubio, 2013; Hijmans, 2016), and used its average in subsequent analyses. We recognize that intertidal habitats can have water and air temperatures that are underestimated by satellite‐based and weather station proxies of air and water temperatures (Lathlean, Ayre, & Minchinton, 2011), and as such, our temperature estimates are indirect proxies of true abiotic niches.

To generate the environmental niche space (termed the PCAenv), we performed a principal component analysis using *ade4* (Dray & Dufour, 2007) on variables that had relatively low correlation (<.9). We defined overlap (O) and expansion (E) populations by comparing the 99% kernel density estimate (*ks*) in the PCAenv of native source populations (i.e., northeastern Japan; Figure 2) and non‐native populations; overlap populations fell within the co‐occurring environmental niche space, while expansion populations are those that did not (Figure 1). In our analyses, we had no populations within unfilled niches (U; sensu Guisan et al., 2014).

### 2.2 Phenotyping

From May until October 2015, we collected live thalli at each of 40 populations: 15 Japanese (six source and nine nonsource), five western North American, 10 eastern North American, and 10 European populations (Figure S1; Table S1). At each site, we haphazardly collected 100 thalli at least 1 meter apart at each site. The water temperatures at the time of collection were statistically indistinguishable between native versus any non‐native region (average = approximately 23°C; Table S1), did not vary with latitude along a continental shoreline, nor predict heat tolerance (analyses not shown). Thalli were shipped to Charleston, SC USA, in small polyethylene bags with seawater‐moistened paper and maintained in the dark at 15–20°C for 3 to 5 days. We then hydrated thalli for 24 hr within liter‐sized plastic containers with seawater collected at high tide in Charleston Harbor (27–30 ppt; 6.8–7.8 pH) placed in a temperature‐controlled incubator (15C). After 12–24 hr, 1‐cm apices were isolated from all thalli and incubated for 12–24 hr at 15°C with a 12‐hr light/dark cycle before initiation of tolerance assays.

We exposed a haphazardly chosen set of approximately 20 thalli per population to extreme heat (1, 2, and 4 hr in 40°C), extreme cold (45, 75, and 105 min at −20°C), and low salinities (8 days at 0, 5, and 10 ppt). The low salinities are within the range that most intertidal, estuarine species typically experience (e.g., Table S1; Weinberger, Buchholz, Karez, & Wahl, 2008). During the summer of 2015 (mid‐May to mid‐September), HOBO^®^ loggers placed in the mid‐intertidal found that southeastern Japan (hik) and the southeastern United States (fjs) mudflats recorded temperatures greater than 40°C for at least 30–120 min during 4–5 days, but temperatures never exceeded 40°C at two native source populations (mou, mng). The cold temperatures we assayed allow us to infer tolerance to freezing tissues; we recognize these interpretations are tempered by the fact that some natural *G. vermiculophylla* populations experience freezing conditions (<0°C) but are unlikely to experience −20°C itself.

*Gracilaria vermiculophylla* populations are typically composed of a mix of haploids and diploids, and both are capable of clonal fragmentation (Krueger‐Hadfield et al., 2016). In order to minimize the influence of including genetically identical clones and a mix of ploidies on our interpretation, we attempted to phenotype only diploids using two methods. First, we did not phenotype reproductive haploid thalli, as these can be readily identified by eye. Second, we used multilocus genotyping methods to remove clones and nonreproductive haploids after phenotyping assays were completed (see Methods: Statistical Analysis).

For the heat tolerance assay, four apices per thallus were individually placed into unsealed 2‐ml microcentrifuge tubes containing 175 μl of seawater. A control apex was kept in a 15°C growth chamber, while three apices were placed in a 40°C water bath for 1, 2, and 4 hr. For the cold tolerance assay, four apices were independently placed into sealed 250 μl PCR tubes containing 200 μl seawater. A control apex was kept in a 15°C incubator, while three apices were placed in a −20° C freezer for 45, 75, and 105 min. Apices were then placed into randomly assigned wells of 12‐well tissue culture plates (2.21‐cm‐diameter wells) filled with 4 ml of seawater and incubated at 15°C for 8 days with a 12‐hr light/dark cycle. For the salinity tolerance assay, four apices were independently placed directly into the 12‐well tissue culture plates filled with 4 ml of seawater at a titration of salinities diluted to 0, 5, and 10 ppt, with 30 ppt serving as a control.

Every 2 days for 8 days, we changed water and assessed thallus bleaching (i.e., loss of photosynthetic pigments; Figure S2) as a proxy of mortality, itself an important component of fitness. Pigment loss is a common stress response for macroalgae (Davison & Pearson, 1996), and completely bleached thalli are dead. We generated a Bleaching Score (BS), where “1” indicated no bleaching, “2” indicated partial bleaching or color change to pink or white, and “3” indicated full bleaching. An analysis with a subset of apices indicated that BS inversely relates to Photosynthetic Quantum Yield of PSII photochemistry, as measured by the Fv/Fm ratio of pulse‐amplitude modulation (PAM) fluorometry (Figure S2).

Remaining portions of the thalli that were unexposed to the initial stressors were reared within a common‐garden environment for 14–22 weeks (average = 17 weeks) in order to minimize the effects of environmental history. Thalli were reared individually within polypropylene tubes (3 cm diameter X 9 cm in length) that had both ends covered with a window screen to prevent thallus escape but enable water exchange. Tubes were randomly assigned to buckets with approximately 15 L of seawater, tumbled rapidly using aquarium pumps and maintained at room temperature (20–23°C), and an approximately 12‐hr dark/light cycle under fluorescent light of approximately 90 μmol m^−2^ s^−1^. Their newly grown apices were then assayed for heat and low‐salinity tolerance as described earlier. In total, we phenotyped 935 field‐collected and 325 common‐garden thalli.

### 2.3 Statistical analysis

We identified and removed clones and haploids by genotyping field‐collected thalli from each location (*n* = 16) at ten microsatellite loci (Table S2). We did not genotype the common‐garden thalli due to a lack of sufficient material. Isolation of genomic DNA, microsatellite simplex PCRs, electrophoresis, and assessment of clonality and *P*_*sex*_ were performed following Kollars et al. (2015) and Krueger‐Hadfield et al. (2016). Clones were determined by calculating *P*_*sex*_ using *RClone* (Bailleul, Stoeckel, & Arnaud‐Haond, 2016), and the ploidy of a thallus was assigned based on the presence of at least one heterozygous microsatellite locus (i.e., diploid, Krueger‐Hadfield et al., 2016). Across the 36 populations that were genotyped and phenotyped, all 16 thalli were diploids in 27 populations. Within only three populations were there four or more haploids (Table S2). In 34 of 36 locations, we detected at least six unique diploid thalli (Table S2). Phenotypic results were analyzed after all haploids and clonal replicates were removed.

The ordinal Bleaching Score (BS = 1, 2 or 3) on day 8 was converted to a Standardized Bleach Score (or SBS = BS_TRT_ – BS_CTRL_) that calculates the difference in BS per thallus at one treatment level relative to the control. Thus, SBS ranged from 2 to −2, although negative SBS values were rare (<1% across all replicates). We generated Analysis of Deviance tables using ordinal logistic regressions (OLR) by implementing a cumulative link mixed model (*clmm*) routine of the *ordinal* package (Christensen, 2015). All OLRs evaluated effects on the response variable (SBS), treated population as a random intercept, and used a likelihood ratio test to generate *p*‐values.

Ordinal logistic regressions assessed the additive and interactive effect of region (native versus non‐native) and treatment (e.g., heat stress). “Native” populations were those within the source region from Japan, as this region is most relevant for assessing phenotypic change sharing evolutionary history. We visualized “Proportion Bleached” as the proportion of apices in a population that had SBS greater than zero.

Ordinal logistic regressions tested for the predictive ability of PC1, PC2, or both axes of the PCAenv to explain the stress tolerance results. OLRs also assessed whether niche shift predicted phenotypic shifts by comparing the phenotypes of overlap populations (O) versus expansion populations (E). In this latter analysis, we did not include northwestern North America populations (moo, ptw, eld) because only ~75% of these thalli originated in northeastern Japan (Figure 2). The surface fit of the phenotypic results was visualized using *fields* (Nychka, Furrer, Paige, & Sain, 2015).

In order to analyze geographic clines in temperature, we employed OLRs to evaluate population‐specific heat tolerance by sea surface temperature, region, or both in an ANCOVA‐like design. Maximum monthly surface seawater temperature (SSTmax; BioOracle) inversely correlates with latitude in Japan (*R* ^2^ = .907) and eastern North America (*R* ^2^ = .914) but has no relationship with latitude along western European coastlines (*R* ^2^ < .001). We visualized the model predictors using the closely related Cumulative Link Models (*clm*) routine. All *R* analyses used version 3.3.3 (R Core Team 2017).

## 3 RESULTS

### 3.1 Niche shift analyses

The first two axes of a principal component analysis of abiotic variables (termed the PCAenv) explained 71% of variation in climate data from all realized and potential habitats (Figure 3b, Table 1). The first axis (42%) was best explained by a negative correlation with mean sea and air temperatures, while the second axis (29%) was best explained by a positive correlation with among‐month variation in sea and air temperature.

**Table 1:** Correlation and relative contributions of bioclimatic variables with PCA axes

Variable	PC1	PC2	PC3	PC4	PC5
Salinity (SAL)	−.535	−.253	.043	.788	−.155
SATmax	−.762	.578	.048	−.059	−.198
SATmean	−.940	.060	.244	−.146	−.111
SATmin	−.858	−.398	.262	−.137	−.015
SATrange	.385	.851	−.252	.107	−.126
SSTmax	−.682	.687	−.142	−.091	−.108
SSTmean	−.976	.134	−.026	−.074	−.032
SSTmin	−.922	−.333	.039	−.027	.093
SSTrange	.306	.902	−.158	−.050	−.181
PREC‐01	.106	−.736	−.476	−.155	−.343
PREC‐04	−.466	−.144	−.819	−.041	.191
PREC‐07	−.478	.563	−.547	.137	.288
PREC‐10	.024	−.475	−.810	−.016	−.145
Total	42.0%	29.3%	16.1%	5.7%	3.1%
Cumulative total	42.0%	71.3%	87.4%	93.1%	96.2%

Gray highlights the two variables with greatest contribution to the PC axis.

PREC, precipitation; SAT, surface air temperature (°C); SST, sea surface temperature (°C).

Based on this PCAenv, we quantified niche shifts by comparing all native (Japan, South Korea, Russia, and China) versus non‐native habitats. On average, non‐native populations seemed to expand into habitats with colder temperatures and less seasonal variation in temperature relative to the full native range (see niche shift indicated by arrows of Figure 3c). However, *G. vermiculophylla* originated from the Pacific coastline of northeastern Japan, a region of Japan that is colder and more seasonal than other, lower‐latitude populations in the native range (see inset in Figure 3a). When we adjusted the niche shift model to compare native source versus non‐native habitats, we find non‐native populations expanded into warmer and less seasonal habitats (Figure 3d), a result that sharply contrasts with the colder temperature expansion inferred from incorporating the entirety of the native range.

### 3.2 Stress tolerance of native source versus non‐native populations

**Figure 4:**
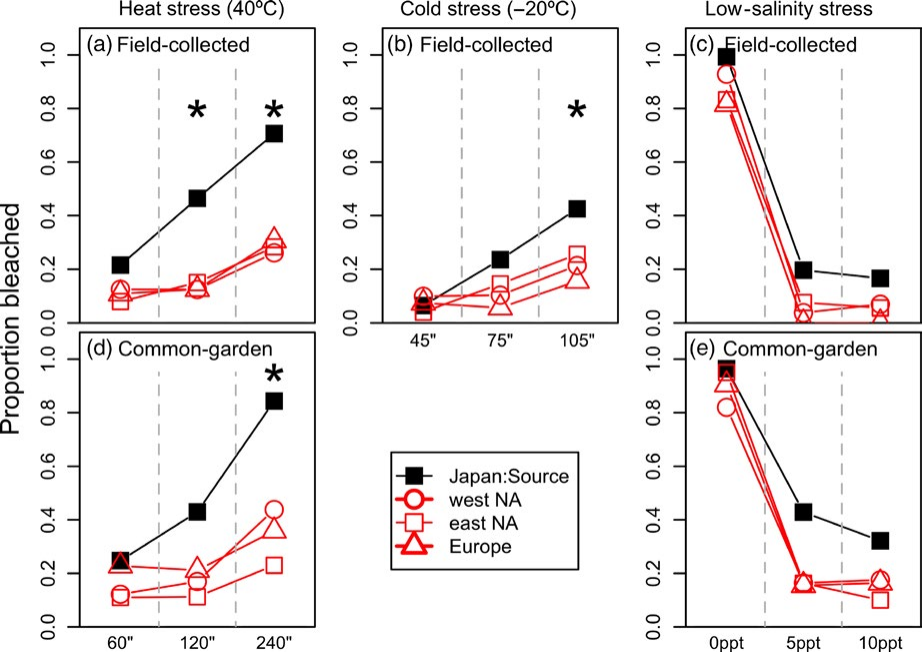
Average proportion of bleached thalli from Japanese source populations (black) versus non‐native populations (red) after exposure to heat (a, d), cold (b), and low salinities (c, e). Thalli were assayed 1 week (“Field‐collected”; a‐c) and approx. 17 weeks (“Common‐garden”; d‐e) after collection. Asterisks indicate treatments in which Japan source populations bleached more frequently than did non‐native populations (Table 2). “NA” indicates North America

This shift into warmer habitats qualitatively predicts that, on average, non‐native populations of this taxon will be more tolerant of elevated temperatures. When exposed to 40°C, field‐collected populations from the introduced range had fewer bleached thalli (i.e., higher survivorship) than did native source populations (Figure 4a; Table 2A). *Post hoc* analyses indicated that non‐native populations had greater survivorship when exposed to 40°C for two and 4 hr, but not after 1 hr. Similarly, after growing thalli in a common environment (Figure 4d; Table 2B), non‐native populations had greater survivorship than did thalli from Japanese source populations when exposed for 4 hr, but not for 1 and 2 hr. The consistency of the field‐collected and common‐garden patterns strongly suggests a genetic component to the population‐level differences.

**Table 2:** Analysis of deviance tables for the effect of treatment, regional source, and their interaction on standardized bleaching probability (SBS) for A, B) heat, C) cold, and D, E) low‐salinity stresses

	d f	All non‐native	wNA	eNA	Eur	df	All non‐native	wNA	eNA	Eur
	A. Heat stress (40°C)—Field‐collected					B. Heat stress (40°C)—Common‐garden				
Overall model										
(1 | population)	1	<.001	<.001	<.001	<.001	1	<.001	<.001	<.001	<.001
Overall model	5	<.001	<.001	<.001	<.001	5	<.001	<.001	<.001	<.001
Region	1	<.001	.008	<.001	<.001	1	<.001	.006	.005	.001
Treatment	2	<.001	<.001	<.001	<.001	2	<.001	<.001	<.001	<.001
Interaction	2	.001	.096	.002	.014	2	<.001	<.001	.001	.001
Post hoc results										
1 hr		.223	.401	.498	.253		.128	.283	.408	.314
2 hr		<.001	.004	<.001	<.001		<.001	.015	.019	.003
4 hr		<.001	.008	<.001	<.001		<.001	.001	.001	<.001
Sample size										
Japan		43 (6)	43 (6)	43 (6)	43 (6)		29 (2)	29 (2)	29 (2)	29 (2)
Non‐native		132 (22)	21 (4)	58 (9)	53 (9)		239 (25)	59 (5)	91 (10)	89 (10)
	C. Cold stress (‐20°C) ‐ Field‐collected									
Overall model										
(1 | population)	1	<0.001	<.001	<.001	<.001					
Overall model	5	<.001	<.001	<.001	<.001					
Region	1	.004	.043	.038	.007					
Treatment	2	<.001	<.001	<.001	<.001					
Interaction	2	.021	.133	.104	.022					
Post hoc results										
45”		.413	0.653	0.335	0.620					
75”		.085	0.232	0.500	0.031					
105”		<.001	0.014	0.003	<0.001					
Sample size										
Japan		83 (6)	83 (6)	83 (6)	83 (6)					
Non‐native		231 (24)	38 (5)	100 (10)	93 (9)					
	D. Low salinity—Field‐collected					E. Low salinity—Common‐garden				
Overall model										
(1 | population)	1	.002	.398	<.001	.010	1	<.001	.887	<.001	.004
Overall model	5	<.001	<.001	<.001	<.001	5	<.001	<.001	<.001	<.001
Region	1	<.001	.014	.007	<.001	1	<.001	.001	.003	<.001
Treatment	2	<.001	<.001	<.001	<.001	2	<.001	<.001	<.001	<.001
Interaction	2	.465	.225	.280	.382	2	.802	.430	.047	.440
Post hoc results										
0 ppt		.015	.166	.024	.013		.004	.128	.009	.003
5 ppt		.004	.019	.079	.003		.004	.047	.119	.005
10 ppt		.006	.243	.054	.020		<.001	.067	.019	.001
Sample size										
Japan		71 (6)	71 (6)	71 (6)	71 (6)		21 (2)	32 (5)	32 (5)	32 (5)
Non‐native		210 (21)	38 (5)	99 (10)	90 (9)		243 (25)	60 (5)	93 (10)	90 (10)

A, C, D represent field‐collected plants, and B, E, represent common‐garden thalli. Models and post hoc tests evaluated Japanese source versus non‐native individuals. We also compared source against western and eastern North America (wNA, eNA) or Europe (Eur). Field‐collected analyses were performed after removing haploids and clones. Sample size for thalli and populations (in parentheses) is also shown. Significant *p*‐values are in gray.

Non‐native populations were also more tolerant of cold stress and low‐salinity stress. Non‐native populations had greater survivorship than did Japanese source populations when exposed to −20°C for 105 min, but not 45 or 75 min (Figure 4b; Table 2C). Non‐native populations had generally greater survivorship than did Japanese source populations across all low‐salinity conditions in both field‐collected and common‐garden experiments (Figure 4c,e; Table 2D,E). When we analyzed continental shorelines (western and eastern North America; Europe) as independent replicates of phenotypic evolution, the patterns of tolerance largely mirrored patterns using all shorelines together (Figure 4; Table 2).

### 3.3 Environmental predictors of heat stress tolerance

**Figure 5:**
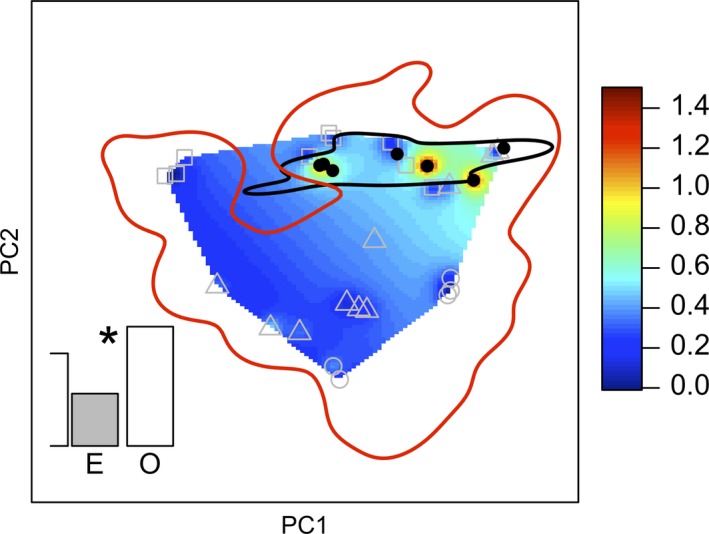
Genetically informed niche shift analysis predicts heat tolerance. Populations within the Overlap (O) niche bleach (i.e., within the black polygon) more frequently bleach than do populations in the Expansion (E) niche (i.e., inside the red polygon excluding the black polygon) after exposure to 40°C for 4 hr. PCAenv is presented as in Figure 2d. The kriging surface represents population‐specific average bleaching probabilities (SBS), where higher values indicate greater bleaching. Inset: Average SBS of O versus E regions; an asterisk indicates *p* < .05 The *y*‐axis tick mark indicates SBS = 0.5. Key: Black circles = Japan source; gray circles, squares, and triangles = western North America, eastern North America, and Europe, respectively

Environmental data (i.e., PC1, PC2, and both axes from the PCAenv) significantly explain the response to heat stress across all native source and non‐native populations (see surface in Figure 5; Table 3), a pattern consistent with local adaptation. A direct and quantitative test of the utility of the niche shift model to predict phenotype is to compare phenotypes from populations in the Expanded habitats (E) versus those in the Overlap habitats (O). Indeed, populations in Expanded niche were more tolerant of heat stress (i.e., have fewer bleached thalli) than populations in the Overlap niche region (Figure 5; Table 3). There was little to no effect of PCAenv axes nor the OvE comparison on cold tolerance or low‐salinity tolerance (Table 3). The exception is that Axis 2 significantly correlated with cold tolerance (i.e., populations with more seasonal water and air temperatures had lower cold tolerance; data not shown).

**Table 3:** *p*‐values from ordinal linear regressions of phenotype results against PCAenv axes or niche shift metric (Overlap vs. Expanded regions or “OvsE”)

Variable	Axis 1	Axis 2	Axis 1 & 2	OvsE
Heat tolerance				
1 hr at 40ºC	.047	.164	.062	.186
2 hr at 40ºC	.129	.038	.045	.086
4 hr at 40ºC	.175	.109	.133	.027
Cold tolerance				
45” at −20°C	.322	.263	.352	.153
75” at −20°C	.667	.020	.051	.194
105” at −20°C	.942	.123	.304	.067
Salinity tolerance				
0 ppt	.814	.327	.612	.622
5 ppt	.795	.117	.290	.232
10 ppt	.409	.101	.202	.057

All comparisons use native source versus all non‐native populations. Significant *p*‐values are in gray.

**Figure 6:**
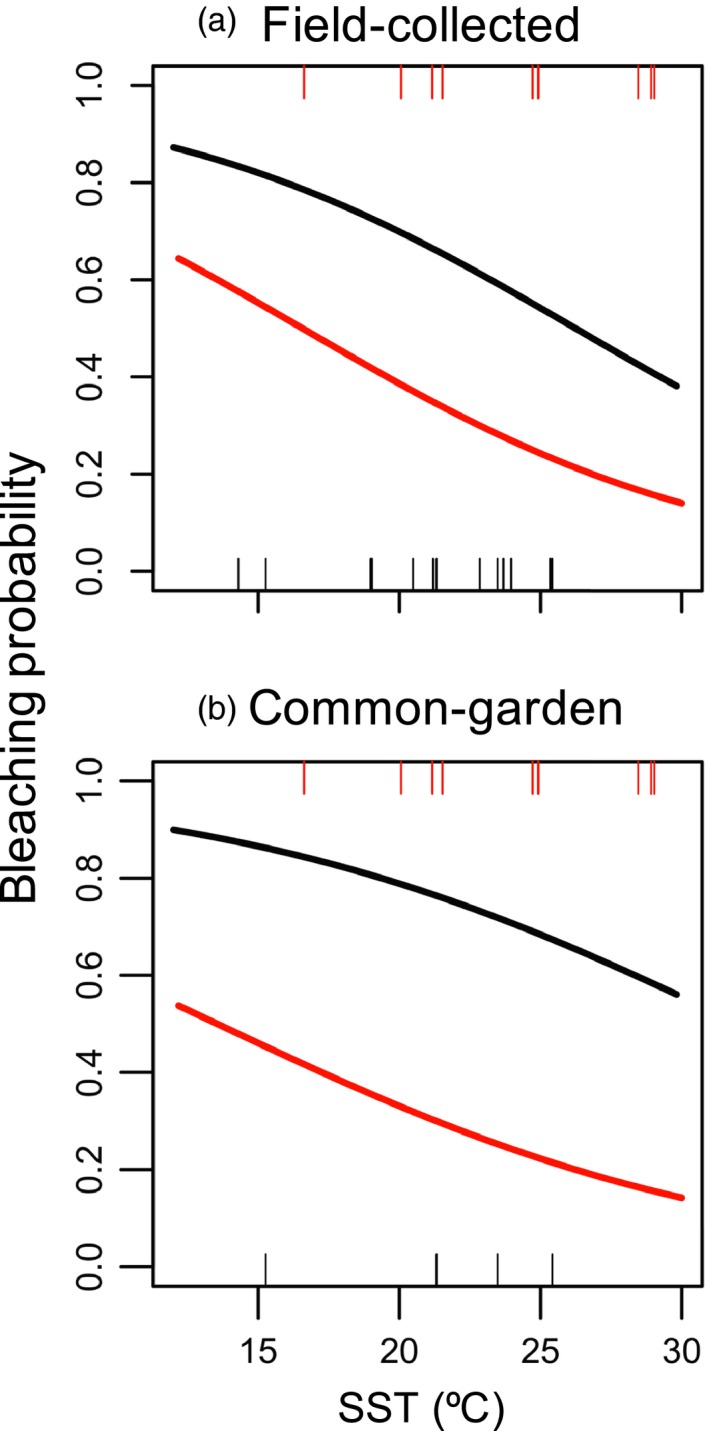
Latitudinal clines of tolerance for heat stress across populations of native (Japan; black) and introduced regions (eastern North America; red). Populations were exposed to heat (40°C) for 4 hr (a) after 1 week (“Field‐collected”) and (b) approximately 17 weeks since collection (“Common‐garden”). Modeled bleaching probabilities are regressed against maximum monthly sea surface temperature (SST)

One clear hallmark of postintroduction adaptation is the generation of a cline along environmental gradients within the introduced range (Moran & Alexander, 2014). Consistent with such local adaptation, eastern North American thalli collected at warmer, low‐latitude sites were more tolerant to heat stress than thalli collected at colder, high‐latitude sites. This latitudinal decline in heat tolerance recapitulated a parallel decline in native Japan, which includes source and nonsource regions (Figure 6; Table 4). The clines differed in intercept because overall, eastern US populations generally had stronger heat tolerance. A similar cline in heat tolerance was not evident in Europe (Table S3), likely because variation in summer sea surface temperatures was not as broad as that of eastern North America and Japan.

**Table 4:** Analysis of deviance tables for the effect of sea surface temperature (SST), regional source (Japan vs. eastern United States), and their interaction on standardized bleaching score (SBS) when exposed to 40°C at 1, 2, or 4 hr

	*df*	1 hr	2 hr	4 hr
A. Field‐collected				
Overall model				
(1 | pop)	1	.001	.002	<.001
Overall model	3	.748	.003	<.001
Region	1	.625	.003	<.001
SST	1	.480	.092	.044
Interaction	1	.572	.423	.933
Sample size				
Japan		76 (12)	76 (12)	76 (12)
Eastern United States		58 (9)	58 (9)	58 (9)
B. Common‐garden				
Overall model				
(1 | pop)	1	.561	.009	<.001
Overall model	3	.423	.011	.001
Region	1	.123	.004	<.001
SST	1	.905	.223	.033
Interaction	1	.642	.326	.973
Sample size				
Japan		40 (5)	40 (5)	40 (5)
Eastern United States		91 (10)	91 (10)	91 (10)

Field‐collected analyses were performed after removing haploids and clones. Sample size for thalli and populations (in parentheses) is also shown. Significant values are in gray.

Results are presented for A) field‐collected thalli and B) common‐garden thalli.

When cold tolerance of Japan versus eastern North America (Table S4a), and Japan versus Europe was compared (Table S4b), we detected a marginally significant interaction between region and minimum sea surface temperature (SST) in both comparisons. This was explained by a significant decline in cold stress tolerance with latitude in Japan, but not in either non‐native coastline (analyses not shown).

## 4 DISCUSSION

We used a genetically informed niche overlap analysis to show that the average climatic niche of non‐native populations of *G. vermiculophylla* is warmer than that of the native source populations in Japan (Figure 2d). This predicts that populations whose niches overlap with the native source range are more sensitive to elevated heat stress than are populations in these expanded invaded niches. Indeed, non‐native populations are generally more tolerant to the warmer temperatures of the Expanded niche (Figure 4a,d), and populations in the niche expansion habitats had greater tolerance for heat stress than did niche overlap populations (Figure 5).

The role of local adaptation during and after introduction was reinforced by two patterns. First, the elevated stress tolerance seen on multiple continental shorelines seeded by independent introduction events (Krueger‐Hadfield et al., 2017) indicates that local selection during or immediately following introduction likely plays a more important role than does genetic drift. Second, the presence of latitudinal clines along the eastern United States recapitulated an analogous cline in Japan (Figure 6). As with other studies that use clonal propagation (e.g., Galloway, 1995), the consistency in phenotypic responses across field‐collected and common‐garden thalli indicates that population‐level differences likely have a genetic basis, but does not preclude a role for environmental history, phenotypic plasticity, or both. We note that previous studies of population‐level differentiation in *G. vermiculophylla* phenotypes (Hammann, Wang, Boo, Aguilar‐Rosas, & Weinberger, 2016; Hammann et al., 2013; Saha, Wiese, Weinberger, & Wahl, 2016; Wang et al., 2017) assayed populations that were not within the source region and thus could not separate prior adaptation from the signal of rapid evolution during the invasion that we infer here.

In addition to rapid evolution of greater tolerance to heat stress, non‐native populations also showed greater tolerance for cold stress and low‐salinity stress. One genetic explanation for the overall stronger tolerance of non‐native populations may be microevolution of the expression of heat‐shock proteins (HSP), which are known to mediate tolerance for heat, cold, and salinity stress in other species (Sørensen, Kristensen, & Loeschcke, 2003; Swindell, Huebner, & Weber, 2007) including marine invaders (Kelley, 2014; Zerebecki & Sorte, 2011). Data from both protein (Hammann et al., 2016) and RNA (B.A. Flanagan, unpublished data) suggest that non‐native populations induce greater levels of HSP relative to native populations. Moreover, compounds constructed to specifically inhibit HSP70 and HSP90 functioning experimentally reduced the ability of a non‐native population to tolerate elevated heat stress, cold stress, and low‐salinity stress (B.A. Flanagan and K. Campbell, unpublished data). Thus, our working hypothesis is that rapid evolution of greater tolerance for heat stress on warmer non‐native estuaries was mediated by microevolution of HSP expression and that this had a pleiotropic impact on tolerance for low salinities and cold stress. This hypothesis awaits further testing.

There are several nonmutually exclusive hypotheses on the timing and form of selection on stress tolerance. The first is widespread local adaptation, in which selection favored stress‐tolerant genotypes establishing themselves on introduced habitats. This postintroduction adaptation seems most parsimonious because it also explains the latitudinal cline in heat tolerance along eastern North America. Second, stress‐tolerant genotypes were more likely to survive transport from Japan (Hammann et al., 2016). Third, seaweed farmers favored stress‐tolerant strains before introduction, as northeastern Japan hosted the harvest and/or aquaculture of multiple gracilarioid species until the mid‐20th century (Okazaki, 1971). However, the extent to which *G. vermiculophylla* itself was intensively cultivated and subject to artificial selection is unknown. Under any of these evolutionary scenarios, clonal fragmentation, which is the dominant reproductive mode in most of the introduced range (Krueger‐Hadfield et al., 2016), may have facilitated evolution of stress tolerance by propagating favorable combinations of alleles without sexual recombination. Distinguishing among these hypotheses will require a description of the genomic architecture of stress tolerance and historical patterns in allele frequencies among available herbarium samples (e.g., Vandepitte et al., 2014).

If the evolutionary response occurred during or after the invasion, it proceeded at a rapid pace. Based on existing evidence that the invasion originated within the last 100 years (Krueger‐Hadfield et al., 2017), our estimates of phenotypic change in heat, cold, and salinity tolerance are 8773, 5697, and 6628 Darwins (sensu Haldane, 1949), respectively (averaged across the three coastline invasions and treatment levels), an evolutionary rate greater than the median of the ~2900 estimates of phenotypic change reported to date in plants or animals (Westley, 2011; Figure S3). Whether phenotypic evolution and its speed seen for *G. vermiculophylla* is exceptional among marine species remains unclear, as the eco‐evolutionary dynamics of nearly all other marine invaders remain undescribed (Tepolt, 2015).

There is an emerging willingness to integrate genetic, experimental, and modeling approaches in understanding invasions. Some studies have combined genetic identification of source populations with niche shift models (e.g., Chefaoui & Varela‐Álvarez, 2017; Chifflet et al., 2016; Fitzpatrick, Weltzin, Sanders, & Dunn, 2007; Ikeda et al., 2016) or with estimates of phenotypic shifts (e.g., Agrawal et al., 2015; Bossdorf et al., 2008; Schrieber et al., 2016). Others have combined tests of phenotypic shifts with niche models (e.g., Dlugosch et al., 2015; Turner, Fréville, & Rieseberg, 2015; Wittmann, Barnes, Jerde, Jones, & Lodge, 2016), but more rarely are all three approaches used simultaneously (but see Rey et al., 2012; Hill, Chown, & Hoffmann, 2013). Here, we show that the simultaneous incorporation of population genetics into niche shift analyses has the ability to predict phenotypic evolution (Guisan et al., 2014; Hill et al., 2013; Van Kleunen, Dawson, Schlaepfer, Jeschke, & Fischer, 2010) and thus will help to quantify when and where rapid adaptation accompanies invasion events. Our results also suggest that studies which compare the entirety of the native and non‐native range may underestimate the strength of evolutionary change, mistakenly infer its direction, or both.

## AUTHOR CONTRIBUTIONS

A.B., B.A.F., C.D., E.E.S., F.W., H.E., M.K., M.N., M.V., P.B., R.T., S.A.K.H., and S.S. collected samples; A.B., B.A.F., E.D., L.L., P.B., and S.S. phenotyped samples; B.A.F. and S.A.K.H. extracted DNA; S.A.K.H. genotyped loci; E.E.S. and S.A.K.H. performed genetic analyses; A.E.S., C.M., E.E.S, and S.A.K.H. designed the study; and E.E.S. analyzed phenotype data and wrote the manuscript. All authors discussed and edited the manuscript.

## CONFLICT OF INTEREST

The authors declare no competing financial interests.

## DATA ARCHIVING STATEMENT

Data are available from the Dryad Digital Repository: https://doi.org/10.5061/dryad.f8c4f
