# Supplementary material for: Combining niche shift and population genetic analyses predicts rapid phenotypic evolution during invasion
Source: Evol Appl. 2018 Feb 2;11(5):781–93. doi: 10.1111/eva.12592 (PMC5978718; doi:10.1111/eva.12592)
Supplement: Supplementary file 2 [file EVA-11-781-s002.docx]

Figure B1. Native and non-native populations of *Gracilaria vermiculophylla* that were phenotyped from A) Japan, B) western North America, C) eastern North America, and D) Europe. See Table B1 for site abbreviations.


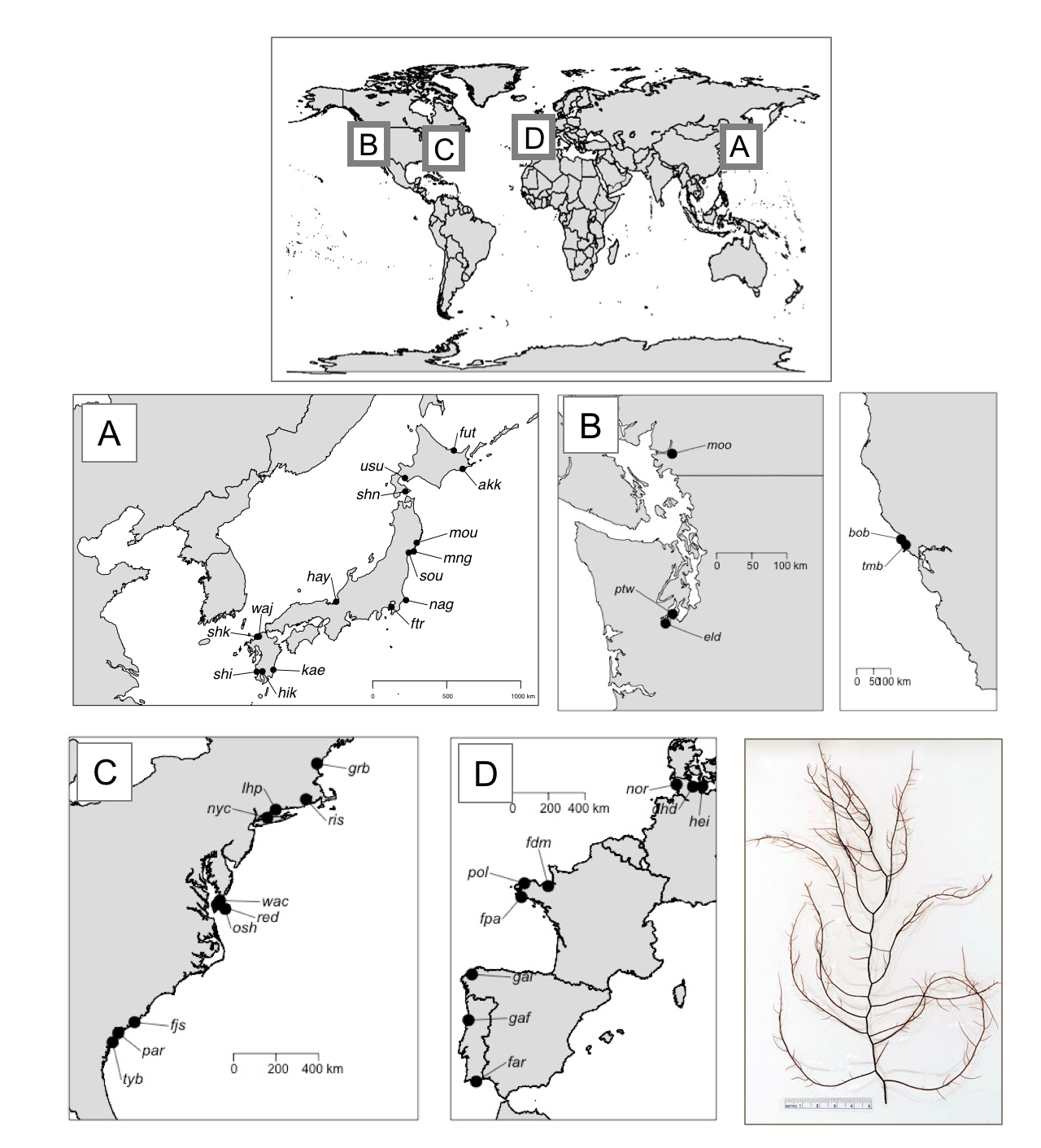


Figure B2. Bleaching score reflects photosynthetic efficiency. *G. vermiculophylla* apices were dark-adapted between 45 minutes and 4 hours before their base value of fluorescence (F_0_) and maximum value (F_m_) were quantified using a JUNIOR-PAM (Walz). Bleaching Score (BS) of “1” indicated no bleaching, “2” indicated partial bleaching or color change to pink or white, and “3” indicated full bleaching. Variation of moderately bleached thalli (BS = 2) likely reflects the spatial scale of bleaching: some cells of that thallus are functional, while neighboring cells have either lost pigment or photosynthetic function.


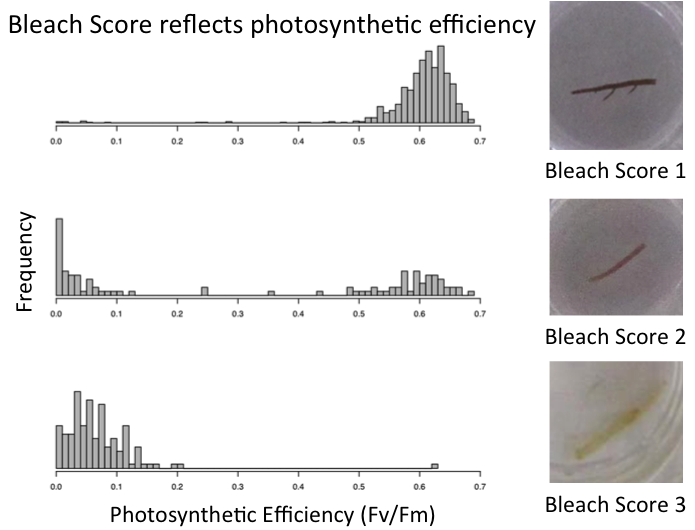


Figure B3. A comparison of Darwins for a variety of animal and plant studies (33, 34) with the average values for the microevolution of *G. vermiculophylla*, assuming 100 years of microevolution. Darwins were calculated as ln(Z_0_*Z_1_^-1^)*(Dt*10^-6^)^-1^, where Z_0_ and Z_1_ = phenotype values in the native source and non-native regions, respectively, and Dt = time in years. 1-Heat stress; field-collected. 2-Cold Stress; field collected. 3-low salinity stress; field collected. 4-Heat stress; common-garden; 5-low-salinity stress; common-garden.

Table B1. Metadata for sites. Abbreviations: Populations were assayed with field-collected tissue (FC) and common-garden tissue (CG). eastNA = eastern North America. westNA = western North America. Source vs Non-Source refer to populations in Japan.

| SiteID | SiteName | Region | Latitude | Longitude | Collection date | Water temperature (ºC) | Salinity (ppt) | FC/CG | Collectors |
| --- | --- | --- | --- | --- | --- | --- | --- | --- | --- |
| akk | Akkeshi Bay | Source | 43.025130 | 144.879657 | 2-Jun-15 | 22.19 | 31 | FC/CG | EE Sotka, SA Krueger-Hadfield, CR Hadfield |
| gaf | Aveiro Portugal | Europe | 40.637814 | -8.665855 | 29-Sep-15 | 25.71 | – | FC/CG | EE Sotka |
| bob | Bodega Bay | westNA | 38.317774 | -123.055447 | 14-Aug-15 | 14.93 | 34 | FC/CG | SA Krueger-Hadfield, N Kollars |
| fjs | Fort Johnson | eastNA | 32.751149 | -79.901191 | 10-Jul-15 | 31.72 | 29 | FC/CG | SA Krueger-Hadfield, BA Flanagan, A Gaffney, SJ Shainker |
| nag | Choshi Nagasaki Tyou | Non-Source | 35.701004 | 140.863333 | 17-Jun-15 | 28.76 | 29 | FC | SA Krueger-Hadfield, CR Hadfield |
| dhd | Heikendorf Kiel | Europe | 54.363825 | 10.193674 | 31-Aug-15 | 21.38 | 15 | FC/CG | SA Krueger-Hadfield, EE Sotka, F Weinberger |
| hei | Heiligenhafen | Europe | 54.380225 | 10.983212 | 31-Aug-15 | 19.33 | 15 | FC/CG | SA Krueger-Hadfield, EE Sotka, F Weinberger |
| nor | Nordstrand | Europe | 54.486315 | 8.812739 | 30-Aug-15 | 20.45 | 27 | FC/CG | SA Krueger-Hadfield, EE Sotka, F Weinberger |
| par | Elliot's Beach | eastNA | 32.317566 | -80.703792 | 2-Sep-15 | 18.27 | 34 | FC/CG | SA Krueger-Hadfield, J Holloway, SJ Shainker, K Benes |
| eld | Eld Inlet | westNA | 47.084893 | -122.978715 | 1-Sep-15 | 18.71 | 22 | FC/CG | EE Sotka, T Mumford |
| far | Faro | Europe | 37.027824 | -8.022425 | 6-Jul-15 | 24.21 | 38 | FC/CG | EE Sotka, A Engelen |
| fdm | Mordreuc Dinan | Europe | 48.514599 | -1.969800 | 12-Aug-15 | 35.58 | 8 | FC/CG | SA Krueger-Hadfield, C Destombe |
| fpa | Pont-l'Abbé | Europe | 47.866948 | -4.215873 | 9-Sep-15 | 25.93 | 5 | FC/CG | SA Krueger-Hadfield, ML Guillemin, C Destombe, M Valero |
| pol | St Pol de Leon | Europe | 48.675775 | -3.971613 | 11-Sep-15 | 16.76 | 2 | FC | SA Krueger-Hadfield, S Mauger |
| ftr | Shintomi Unga Futtsu | Non-source | 35.321766 | 139.834452 | 14-Sep-15 | 19.19 | 24 | FC/CG | SA Krueger-Hadfield, CR Hadfield |
| fut | Futatsuiwa Abashiri | Source | 44.049317 | 144.257900 | 11-Sep-15 | 20.43 | – | FC | SA Krueger-Hadfield, CR Hadfield, T Yorisue |
| gal | A Coruña | Europe | 43.328288 | -8.378920 | 8-Sep-15 | 18.4 | – | FC/CG | EE Sotka, L Couceiro |
| grb | Great Bay | eastNA | 43.092076 | -70.864714 | 13-Sep-15 | 18.39 | 30 | FC/CG | SA Krueger-Hadfield, JE Byers |
| hay | Hayase River | Non-source | 35.615327 | 135.906486 | 4-Jun-15 | 22.62 | 16 | FC | SA Krueger-Hadfield, CR Hadfield, M Kamiya, T Ogawa |
| hik | Hikiyama Kajiki | Non-source | 31.732100 | 130.671644 | 27-Sep-15 | 20.21 | 10 | FC/CG | EE Sotka, R Terada |
| kae | Kaeda Miyazaki | Non-source | 31.817214 | 131.447808 | 11-Jun-15 | 21.32 | 9 | FC/CG | EE Sotka, C Sotka, R Terada |
| lhp | Lighthouse Point | eastNA | 41.248826 | -72.904226 | 23-May-15 | 22.72 | 30 | FC/CG | SA Krueger-Hadfield, M Smylie, A Demko |
| mng | Mangoku-ura. | Source | 38.418195 | 141.413173 | 17-May-15 | 27.08 | 14 | FC | SA Krueger-Hadfield, CR Hadfield, H Endo, J Pocklington |
| mou | Moune Bay | Source | 38.901194 | 141.622668 | 16-Jul-15 | 17.7 | 23 | FC | EE Sotka, H Endo, J Pocklington |
| nyc | Crab Meadow | eastNA | 40.928615 | -73.326850 | 25-May-15 | 35.13 | 27 | FC/CG | SA Krueger-Hadfield, A Blakeslee |
| osh | Hillcrest | eastNA | 37.284019 | -75.409702 | 16-Jul-15 | 25.68 | 32 | FC/CG | SA Krueger-Hadfield, BA Flanagan, S Fate |
| moo | Port Moody | westNA | 49.279999 | -122.851690 | 4-Aug-15 | 26.1 | 22 | FC/CG | EE Sotka |
| ptw | Pt. Wilson | westNA | 47.210495 | -122.842044 | 17-Jul-15 | 27.64 | 22 | FC/CG | EE Sotka, T Mumford |
| ris | Sandy Point | eastNA | 41.661794 | -71.409895 | 17-Jul-15 | 28.76 | 32 | FC/CG | SA Krueger-Hadfield, N Colvard |
| tyb | Tybee Cut | eastNA | 31.950832 | -80.984364 | 9-Jul-15 | 31 | 28 | FC/CG | SA Krueger-Hadfield, PM Bippus, SJ Shainker, A Baumgardner, L Haram, C Kinney |
| shi | Shimabira Kushikino | Non-source | 31.706818 | 130.271885 | 15-May-15 | 25.42 | 4 | FC | EE Sotka, C Sotka, R Terada |
| shk | Shikanoshima | Non-source | 33.663130 | 130.296200 | 18-May-15 | 19.76 | 32 | FC | EE Sotka, C Sotka, R Terada, S. Kawaguchi |
| shn | Shinori Hakodate | Source | 41.764864 | 140.818178 | 31-May-15 | 15.76 | 35 | FC | EE Sotka, SA Krueger-Hadfield, CR Hadfield |
| sou | Soukanzan | Source | 38.352839 | 141.059694 | 13-Jun-15 | – | 33 | FC/CG | SA Krueger-Hadfield, CR Hadfield, H Endo, J Pocklington, EE Sotka |
| tmb | Tomales Bay | westNA | 38.179647 | -122.909344 | 13-Aug-15 | 17.95 | 36 | FC/CG | SA Krueger-Hadfield, N Kollars |
| wac | Wachapreague | eastNA | 37.619458 | -75.669593 | 2-Aug-15 | 32.98 | 35 | FC/CG | SA Krueger-Hadfield, BA Flanagan, S Fate |
| usu | Usu City | Source | 42.515497 | 140.786180 | 8-Jun-15 | 19.57 | 29 | FC | SA Krueger-Hadfield, CR Hadfield |
| waj | Wajiro Fukuoka | Non-source | 33.673563 | 130.426959 | 18-May-15 | 20.33 | 32 | FC | EE Sotka, C Sotka, R Terada |
| red | Willis Wharf | eastNA | 37.455851 | -75.818625 | 3-Aug-15 | 24.5 | 33 | FC/CG | SA Krueger-Hadfield, BA Flanagan, S Fate |

Table B2. The number of thalli that were both phenotyped per population (n) that were diploids, haploids and unique genotypes (as determined by Psex; Bailleul et al. 2016).

| Site_abb | n | Diploids | Haploids | Unique diploid genotypes |
| --- | --- | --- | --- | --- |
| akk | 16 | 14 | 2 | 14 |
| bob | 16 | 16 | 0 | 7 |
| dhd | 16 | 14 | 2 | 14 |
| ebp | 16 | 16 | 0 | 8 |
| eld | 16 | 16 | 0 | 1 |
| far | 16 | 16 | 0 | 12 |
| fdm | 16 | 15 | 1 | 12 |
| fjs | 16 | 15 | 1 | 11 |
| fpa | 16 | 16 | 0 | 11 |
| ftr | 16 | 16 | 0 | 9 |
| fut | 16 | 16 | 0 | 16 |
| gaf | 16 | 16 | 0 | 12 |
| gal | 16 | 14 | 2 | 8 |
| grb | 16 | 16 | 0 | 6 |
| hay | 16 | 16 | 0 | 16 |
| hei | 16 | 16 | 0 | 10 |
| hik | 16 | 11 | 5 | 11 |
| lhp | 16 | 16 | 0 | 15 |
| mng | 16 | 16 | 0 | 15 |
| moo | 16 | 16 | 0 | 9 |
| mou | 16 | 8 | 8 | 8 |
| nag | 16 | 12 | 4 | 12 |
| nor | 16 | 16 | 0 | 15 |
| nyc | 16 | 16 | 0 | 16 |
| osh | 16 | 16 | 0 | 10 |
| pol | 16 | 16 | 0 | 5 |
| ptw | 16 | 16 | 0 | 7 |
| red | 16 | 16 | 0 | 16 |
| ris | 16 | 16 | 0 | 13 |
| shk | 16 | 16 | 0 | 16 |
| sou | 16 | 16 | 0 | 16 |
| tmb | 16 | 15 | 1 | 15 |
| tyb | 16 | 16 | 0 | 8 |
| usu | 16 | 16 | 0 | 16 |
| wac | 16 | 16 | 0 | 12 |
| waj | 16 | 16 | 0 | 16 |

Table B3 Analysis of Deviance tables for the effect of maximum sea-surface temperature (SST). The effect of regional source (Europe versus Japan) and their interaction on Standardized Bleaching Score (SBS) when exposed to 40ºC for 1, 2, or 4 hours. Field-collected analyses were performed after removing haploids and clones. Sample size for thalli and populations (in parentheses) are also shown. Significant p-values from a likelihood-ratio test are in grey.

| **Overall model** | df | 1 hour | 2 hours | 4 hours |  |
| --- | --- | --- | --- | --- | --- |
| (1 \| pop) | 1 | 0.001 | 0.008 | <0.001 |  |
| Overall model | 3 | 0.634 | 0.002 | 0.002 |  |
| Region | 1 | 0.309 | <0.001 | <0.001 |  |
| SST | 1 | 0.911 | 0.383 | 0.060 |  |
| Interaction | 1 | 0.771 | 0.502 | 0.668 |  |
| **Sample size** |  |  |  |  |  |
| Japan |  | 76 (12) | 76 (12) | 76 (12) |  |
| Europe |  | 53 (10) | 53 (10) | 53 (10) |  |
|  |  |  |  |  |  |

Table B4 Analysis of Deviance tables for the effect of minimum sea surface temperature (SST). The effect of regional source and their interaction on Standardized Bleaching Score (SBS) when exposed to -20ºC for 45”, 75” or 105”. A) Japan versus eastern North America and B) Japan versus Europe. Field-collected analyses were performed after removing haploids and clones. Significant p-values from a likelihood-ratio test are in grey.

| A) Japan versus eastern North America | | |  |  |  |
| --- | --- | --- | --- | --- | --- |
| **Overall model** | df | 45" | 75" | 105" |  |
| (1 \| pop) | 1 | 0.596 | <0.001 | <0.001 |  |
| Overall model | 3 | 0.703 | 0.483 | 0.011 |  |
| Region | 1 | 0.427 | 0.395 | 0.037 |  |
| SST | 1 | 0.335 | 0.285 | 0.386 |  |
| Interaction | 1 | 0.835 | 0.550 | 0.011 |  |
|  |  |  |  |  |  |
| B) Japan versus Europe | |  |  |  |  |
| **Overall model** | df | 45" | 75" | 105" |  |
| (1 \| pop) | 1 | 0.171 | <0.001 | <0.001 |  |
| Overall model | 3 | 0.865 | 0.062 | 0.006 |  |
| Region | 1 | 0.898 | 0.009 | 0.006 |  |
| SST | 1 | 0.394 | 0.739 | 0.174 |  |
| Interaction | 1 | 0.976 | 0.593 | 0.051 |  |
|  |  |  |  |  |  |
